# Fluxgate Magnetometers Based on New Physical Principles

**DOI:** 10.3390/s25133893

**Published:** 2025-06-22

**Authors:** Ivan V. Bryakin, Igor V. Bochkarev, Vadim R. Khramshin, Vadim R. Gasiyarov, Ivan N. Erdakov

**Affiliations:** 1 Laboratory of Information and Measuring Systems of the Institute of Mechanical Engineering, Automation and Geomechanics, National Academy of Sciences of the Kyrgyz Republic, Bishkek 720010, Kyrgyzstan; bivas2006@yandex.ru; 2 Department of Electromechanics, Kyrgyz State Technical University Named after I. Razzakov, Bishkek 720010, Kyrgyzstan; elmech@mail.ru; 3 Power Engineering and Automated Systems Institute, Nosov Magnitogorsk State Technical University, 455000 Magnitogorsk, Russia; hvrmgn@gmail.com; 4 Department of Automation and Control, Moscow Polytechnic University, 107023 Moscow, Russia; gasiyarovvr@gmail.com; 5 Department of Metal Forming, South Ural State University, 454080 Chelyabinsk, Russia

**Keywords:** fluxgate magnetometer, permanent composite-conducting ferrite magnet, cut cylindrical metal electrode, polaron harmonic oscillator, ferrite rod waveguide, spin oscillations, spin wave resonance, Helmholtz coils

## Abstract

This article considers a fluxgate magnetometer (FM) that operates based on a new physical principle. The authors analyze how the alternating electric charge potential of a cylindrical metal electrode impacts the structure of a cylindrical permanent magnet made of composite-conducting ferrite. They demonstrate that this impact and permanent magnet structure initiate the emergence of polarons with oscillating magnetism. This causes significant changes in the entropy of indirect exchange and the related sublattice magnetism fluctuations that ultimately result in the generation of circularly polarized spin waves at the spin wave resonance frequency that are channeled and evolve in dielectric ferrite waveguides of the FM. It is demonstrated that these moving spin waves have an electrodynamic impact on the measuring FM coils on the macro-level and perform parametric modulation of the magnetic permeability of the waveguide material. This results in the respective variations of the changeable magnetic field, which is also registered by the measuring FM coils. The authors considered a generalized flow of the physical processes in the FM to obtain a detailed representation of the operating functions of the FM. The presented experimental results for the proposed FM in the field meter mode confirm its operating parameters (±40 μT—measurement range, 0.5 nT—detection threshold). The usage of a cylindrical metal electrode as a source of exciting electrical change instead of a conventional multiturn excitation coil can significantly reduce temperature drift, simplify production technology, and reduce the unit weight and size.

## 1. Introduction

In practice, various physical parameters are controlled using different probes and metering systems [1]. These include magnetometers that are designed to measure magnetic field parameters. Their applications are particularly wide, from geophysical and space research to subsurface probing and non-destructive testing of various objects. They are also used in navigation systems, robotics, medical devices, and various research activities, etc.

There are several magnetometer types that operate based upon different physical principles [2]. Conventionally, there are five main types, as follows:

1. Proton magnetometers (PM) that use nuclear magnetic resonance.
2. Fluxgate magnetometers (FM) that are based on the changes in core parameters due to the magnetic flux impacts.
3. Quantum magnetometers (QM) that use quantum effects when measuring magnetic fields.
4. Resistance magnetometers (RM) that operate using changes in material resistance due to magnetic field impacts.
5. Hall effect sensors (HS) that use the potential difference (Hall potential) proportional to the magnetic force and current that occurs at the edges of the conductor placed inside a magnetic field when the current is passed through it.
6. Optical magnetometers (OM) that use lasers to measure magnetic fields.

Considering the functional capacities, magnetometers can be divided into vector and scalar. Vector magnetometers are used to measure the magnetic induction direction and scalar magnetometers are used to measure the overall magnetic field intensity. The FM is an example of a vector magnetometer, and the QM is an example of a scalar magnetometer.

Magnetometers have wide applications [3,4,5,6], and various magnetometer types can be used to measure magnetic field parameters in each specific case. However, there are also preferred application areas. For instance, FMs are used to measure weak magnetic fields [7], PMs are mainly used in geophysics and archaeology [8,9], QMs are used to study the magnetic field of large territories [10], RMs and HS are used in industries [6,11], and OMs are used in medicine and biology [12].

FMs have several advantages over other magnetometer types. They have a high sensitivity and can detect very weak magnetic fields over a wide temperature range and under electromagnetic interference. Additionally, they can measure both the magnitude and the direction of the magnetic field. They have low noise levels, high reliability and longevity, and are relatively compact, which makes them suitable for magnetic field measurements on Earth or in space, as well as for usage in navigation, orientation, and stabilization systems; geological exploration, magnetic airborne prospecting, the discovery of hidden objects with a large detection range, studying magnetic properties, and performing non-destructive testing of materials and products, etc. FMs can also operate under various natural magnetic anomalies.

However, FMs have several drawbacks associated with the usage of multiturn excitation coils. This results in the following negative effects:

- Greater implementation costs due to more complex coil winding processes, causing greater FM costs compared to magnetometers with simple sensor technologies, e.g., systems based on microelectromechanical (MEMS), magnetoresistive, and magnet induction technology, or the Hall effect;
- Larger and heavier designs of FMs compared to magnetometers based on new technology like MEMS-based magnetoresistive sensors. This can prevent the usage of FMs in systems with strict size and weight requirements, e.g., drones;
- Increased power consumption required for the excitation current generation and control in the primary coil;
- Temperature drift, which may require regular information signal processing system calibration to provide measurement precision.

There are multiple different FM designs that differ in their functional and metrological parameters. Attempts to improve their technical and economic indicators are being made all over the world [13,14,15,16,17,18]. However, it is difficult to achieve a significant improvement in these indicators using conventional approaches to FM design.

Currently, FM improvement involves several aspects, as follows:

- The improvement of core magnetic material parameters, particularly the usage of amorphous alloys;
- The improvement of electronic hardware, e.g., excitation generators and output signal processing devices;
- The improvement of core quality by using new processing methods;
- The optimization of geometry and dimensions;
- Changing the core configuration.

However, note that the design principle of FM magnetic systems is the same. Thus, we suggest considering the possibility of developing an FM operating system based on a new physical principle that can significantly improve the metrological parameters of FMs. This confirms the relevance of this research.

## 2. Physical Processes in the Groundwork of the New FM Development

To justify the suggested new FM development principle using the phenomenological approach, one has to review the operational physical processes features.

In terms of applicability, all materials may have both beneficial and secondary properties. Researchers have to evaluate the possibility of amplifying the beneficial properties and mitigating the secondary ones through the study of their physical nature.

To do this, the authors review various physical factors that determine the possibility of developing functional devices, particularly fluxgate magnetometers based on new physical principles.

There is a large class of magnetically ordered materials, like ferromagnetics and antiferromagnetics, where elementary magnetic moments of atoms, ions, and electrons are ordered, i.e., magnetic moments in the materials are naturally or spontaneously ordered. To achieve this magnetic ordering, the material has to meet the following two conditions: 1, it must have enough transition element atoms or ions, i.e., elements where the magnetic moment **M***_J_* differs from zero due to the lack of electrons in *d* and *f* shells; 2, magnetic atoms must have specific exchange interactions that result in the ordering of their magnetic moments.

In this case, the magnetic moment of an atom is heavily impacted by the electric field of the crystalline lattice (compared to the electrical interaction, the magnet–dipole interaction of atoms in the lattice is very weak) and generated by atoms around a specific magnetic atom. In this case, the electric field of the lattice will affect the orbital trajectory of electrons (the electron cloud of an atom) and, as a result, the orbital moment.

The spin magnetic moment is only affected by the internal crystalline lattice field through the magnetic spin–orbital coupling, whose energy is calculated using the following expression [19]:

**W***_L_*_,*S*_ = λ × [**M***_L_*, **M***_S_*], (1)

where **M***_L_* and **M***_S_* are the orbital and spin moments of the atom, and λ is the spin–orbital coupling constant.

If the interaction energy of the internal crystalline lattice field **W***_K_* with the magnetic field of the atom is significantly lower than the energy **W***_L_*_,*S*_ of the spin–orbital coupling (i.e., magnetic interaction of moments), the interaction of the intercrystalline field and the atom fields will not have a significant impact on its electron orbit and, therefore, the magnetic properties of the atom will be determined by the sum of magnetic moments **M***_L_* and **M***_S_*, i.e., its full magnetic moment **M***_J_*.

Note that when **W***_K_* >> **W***_L,S_*, the internal crystalline lattice field significantly impacts the electron orbit of the atom and “freezes” its orbital magnetic moment **M***_L_*. Due to this, the latter is not involved in the generation of the atom’s full magnetic moment. In this case, the magnetic moment of the atom is almost exclusively determined by the spin magnetic moments of unpaired electrons in the 3D shell. Therefore, magnetically ordered materials may have conduction electrons (collective electrons) as magnetism carriers that have exchange interactions.

The general expression for the exchange interaction energy necessary for the magnetic ordering of solid objects can be written down as follows [20]:

(2) $$W_{exc}=\sum_{ij}A_{ij}\times S_{i}\times S_{j},$$

where *S_i_* and *S_j_* are the spins of the interacting electrons; *A_ij_* is the exchange integral depending on the distances between electrons *r_ij_*.

Thus, the exchange interaction *s*–*d*(*f*) can cause oscillatory polarization of conduction electron spins. Essentially, the exchange predetermines the nature of ferromagnetism, i.e., spontaneous ordering of magnetic moments in ferromagnetic materials. It is electrostatic by nature, and the average energy of the electrostatic (Coulomb) interaction of electrons depends on the mutual orientation of their spin moments. It is at the minimum in ferromagnetics when they are parallel [21].

The indirect exchange interaction via conduction electrons that has a long-range force is of special interest as, at large interatomic distances, it decreases as the third power of distance and oscillates. This interaction is typical of materials containing a sufficient number of free electrons that act as mediators in the interaction. In this case, a magnetic ion induces oscillatory spin polarization of nearby free electrons.

The representation of the exchange interaction *s*–*d*(*f*) is crucial in the exchange mechanism, which states that magnetics feature the following two groups of electrons:

1. Localized electrons of uncompensated *d* or *f* shells of atoms;
2. Collective electrons of valent (*s*, *p*, ...) energy bands responsible for electric properties and contributing the most to magnetism.

Due to the *s*–*d*(*f*) exchange, conduction electron spins are magnetized (polarized) by localized *d* and *f* electrons. Since the *s–d*(*f*) interaction depends on the spin direction, conduction electrons with different spin directions react differently to the *s*–*d*(*f*) exchange.

For instance, electrons with spin *s_i_* oriented similarly to *S_n_* are generally close to the *n* node, while electrons with a reverse spin are removed from it, which, in turn, results in electron density oscillation, i.e., the density of electrons with the set spin direction changes as we move away from atom *n* along the radius *R*. Conduction electron polarization caused by the spin of one 4*f* atom located at point *R_n_* affects the spin of another 4*f* atom at point *R_m_*, i.e., they implement an indirect exchange link between the 4*f* atoms [22].

The dynamic heterogeneity effects in magnetically ordered materials are especially interesting. For instance, functional magnetoelectronics studying magnetoelectronic effects and phenomena in magnetically ordered continuous media use static heterogeneity, i.e., a localized region with different properties on the surface or inside of an object created as a result of specifically defined processes.

Based on the properties of these static heterogeneities that support the generation and control of various electromagnetic processes in continuous magnetically ordered media, there is a real possibility of creating sensor devices using dynamic heterogeneities of a magnetoelectronic nature that are implicitly linked to electrons. In this case, dynamic heterogeneity is a localized volume with different properties on the surface or inside the object that has no static heterogeneities inside of it and is generated as a result of specific physical and chemical processes. Dynamic heterogeneity can be localized or move within the working volume of the continuous object due to the interaction with various physical fields or dynamic heterogeneities of the same or different physical nature. The movement of such dynamic heterogeneities can be complemented by an active field interaction with detector elements of sensors.

These dynamic heterogeneities of various physical nature may include the following:

1. Dynamic wave heterogeneities (surface acoustic waves; static magnetic waves; spatial charge waves; charge density waves; spin waves; etc.);
2. Charge particle and quasi-particle ensembles (charge packets of electrons, fluxons, etc.);
3. Domains (ferroelectric domains, Gunn domains, cylindrical magnetic domains, etc.). In particular, functional magnetoelectronics often use resonances and waves as dynamic heterogeneities.

Additionally, when considering magnetics from the standpoint of physics and symmetry in terms of the full local magnetization vector **M**(*r*) and antiferromagnetism vector **L**(*r*), small linear oscillations of magnetic moments can be described with spin waves (magnons) that include a type referred to as anti-magnons, which typically features oscillations of antiferromagnetism vector **L** and unchanged vector **M**. In contrast to other types of magnons, whose oscillatory variables also include the components of vector **M** that can be excited by an alternating magnetic field **H**(*t*), anti-magnons can only be excited by an alternating electric field **E**(*t*).

In this case, it is crucial to understand the interaction mechanisms between the magnetic system and electric field whose existence is not linked to the magnetic symmetry of the ferromagnetic medium.

Thus, we can identify a low-frequency group of phenomena associated with the oscillations of vector **M** or **L** caused by field **E**(*t*). For instance, if one of the magnetic vectors (**M** or **L**) is constant and determines the basic (lowest-energy) state that we need, field **E**(*t*)∝ехр(−*j*ω*t*) will “sway” the other one (as part of the linear response). At the same time, the aforementioned antiferromagnetic oscillations or antimagnons can be excited in the ferromagnetic phase [23].

For instance, under certain conditions, an alternating magnetic field can excite coherent spin waves with specific frequencies ω, certain wave vectors **k**, and ordered phases whose changes follow the traveling or standing wave law. As a result, the magnetic moments (spins) of electrons are broken. In this case, broken spins scattered over the workpiece act as elementary excitations of magnetically ordered objects. However, these excitations have wavelike properties, i.e., they are characterized by specific wave vectors **k** and frequencies ω, and are essentially spin waves.

According to the above, we can assume that there can be a physical effect of the alternating potential of a heterogeneous electric field affecting the oscillatory nature of conduction electron spin polarization in metal and magnetically ordered structures. In other words, alternating electric field potential is an external factor that can be used to make targeted impacts on the polariton dynamics of magnetically ordered structures.

Consider the interaction with a heterogeneous alternating electric field generated by electric charges *q_i_*, with alternating potential φ*_i_* distributed along a spatial circle and the structural elements of a conducting permanent magnet (PM).

**Figure 1:**
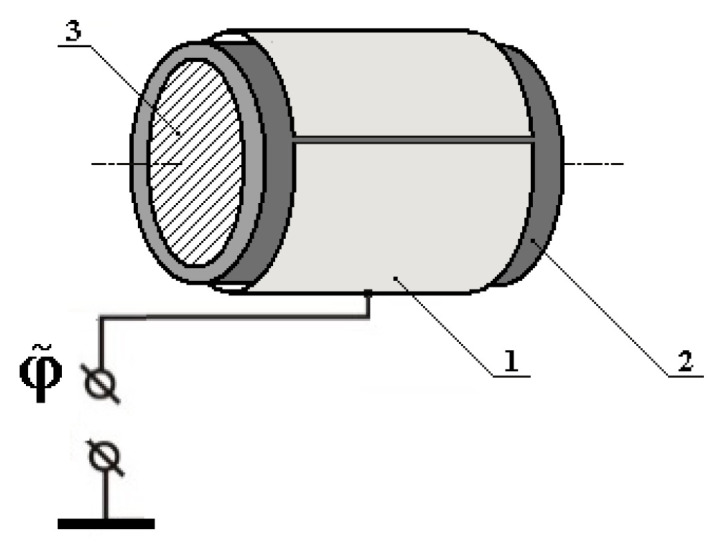
Interaction effect excitation module.

In this case, we implement an interaction between the electric potential $\tilde{\varphi }$ of the electric charge, which is spatial-distributed across the surface of a thin-wall metal electrode (ME) 1, made as a cut hollow cylinder with a finite length and featuring the elements of the PM 3 structure. It is a short cylinder made of conducting ferrite, positioned coaxially inside the cut ME 1 (Figure 1). PM 3 and ME 1 have a galvanic decoupling made with a dielectric cylindrical spacer 2 located between them.

A harmonic signal generator is used to generate electric excitation charges $\pm q_{Ei}(t)$, with respective alternating electric potentials distributed across the surface of a thin-wall cylindrical ME, as follows:

(3) $$\pm \varphi_{Ei}(t)=k\times q_{Ei}(t)/r_{i},$$

where $k={\left(4\times \pi \times \varepsilon_{0}\right)}^{-1}$.

Distributing electric excitation charges $\pm q_{Ei}(t)$, in turn, induce distributed opposite-sign electric charges $\mp q_{Ii}(t)$ on the cylindrical surface of the conducting ferrite PM with respective alternating electrical potentials, as follows:

(4) $$\pm \varphi_{Ii}(t)=k\times q_{Ii}(t)/r_{i}.$$

**Figure 2:**
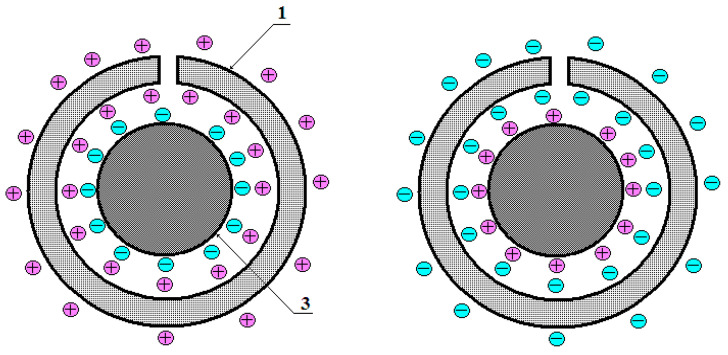
Electric charge redistribution options.

During each change semi-period of the electric potential supplied from the respective generator, conduction electrons and positive ions are redistributed until the electric potentials of the conduction electron and positive ion field compensate for the alternating electric potential of the external field inside the conducting PM material (Figure 2).

The projection of field *E_ri_* intensity vector on the radial movement *dr_i_* of charge *q_i_* in the Cartesian coordinate system can be expressed as follows:

(5) $$E_{ri}=-r_{i}^{0}\times \left(\frac{\partial \phi_{i}}{\partial r_{i}}\right)=-\mathrm{grad}\varphi_{i}=-\nabla \varphi_{i},$$

where ∇ is the Hamilton operator.

In this case, the motion equation for charge *q_i_* (electron) under the alternating electric field with the intensity *Ε_ri_* = *E*_0_ × ехр (*j*ω*t*) can be written down as follows:

(6) $$m_{ei}\times \frac{d^{2}r_{i}}{dt^{2}}=-e\times E_{ri}\times \exp(j\omega_{0}t),$$

where *r_i_* is the coordinate oriented parallel to the electric field *E_ri_*; *m_ei_* is the electron mass.

Having solved Equation (6), we obtain the following result:

(7) $$r_{i}=E_{ri}/(m_{ei}\times \omega_{0}^{2}).$$

The radial displacement of the electron is accompanied by the emergence of a dipole moment −*e_i_* × *r_i_*. Considering this, the magnetizations of a PM structure unit can be expressed as follows:

(8) $$M_{i}=-N_{i}\times e\times r_{i}=\frac{N_{i}\times e^{2}}{m_{ei}\times \omega_{0}^{2}}\times E_{ri}$$

where *N_i_* is the concentration of free electrons in the PM structure oriented in the direction $r_{i}^{0}$.

An electric field does not affect the magnetic spin moment of a fixed charge. The motion of a particle in the electric field is accompanied by the emergence of a magnetic field in the coordinate system of the particle due to the Lorentz transformation. In this case, the following expression is true for the interaction represented by the Lorentz force between the electron moving at velocity **v***_e_* and the magnetic field with induction $B_{P}$ generated by the magnetic moments of electrons **p***_e_*:

**F***_L_* = e × [**v***_e_*, **B**_μ_], (9)

where $B_{p}=\mu_{0}\times \frac{3n\times (p_{e}\cdot n)-p_{e}}{r^{3}}$; *r* is the distance from the electron to the point in which the field is determined, **n** is the single vector with direction r; and *n* is the concentration of free electrons.

In this case, the electron energy level is split, and magnetic-field-wise and counter-magnetic-field-wise spin states emerge.

Note that there is a magnetic interaction between electrons due to the interaction of their magnetic moments, as follows:

(10) $$F_{p}=-\mathrm{grad}(p_{e}\times B_{p}).$$

To sum up, we can claim that the effect in question can regularly initiate the oscillatory redistribution of electrons and positive ions in the PM structure in the radial direction. This ultimately forms the areas with different electric charge densities and the respective alternating electric potential within the conducting material of PM 3.

Consider the classification of magnetic field value that can affect various physical processes, as follows:

1. *p* × *H*_1_~*k* × *T*, where *p* is the magnetic moment of the electron or the atom, *k* is the Boltzmann constant, and *T* is the temperature.

A field exceeding *H*_1_ can have a significant impact on the balanced orientation of electron or atom spins.

2. *p* × *H*_2_~|*W_a_*|, where *W_a_* is the atom and molecular term energy, as follows:

(11) $$W_{a}=-m_{e}\times c^{2}\times {\left(\frac{e^{2}}{\hbar c}\right)}^{2}.$$

A field exceeding *H*_2_ can have a significant impact on the structure of atoms, their ionization energy, and molecular link energy.

3. There is a characteristic field *H*_3_ demonstrating that

(12) $$\frac{\hbar }{m_{e}\times c}\times H_{3}=m_{e}\times c^{2}.$$

This characteristic field changes the properties of the vacuum and, in particular, affects the propagation of electromagnetic waves in the vacuum.

The analysis of the classification above shows that the values of fields *H*_2_ and *H*_3_ do not allow for the replication of these fields in laboratory conditions on Earth. Thus, field *H*_1_ is of special practical interest.

Following the notes made, analyze the specifics of the physical processes that occur during the cyclic redistribution of conduction electrons and positive ions in the conducting PM structure.

The stability of metal lattices formed by positive ions is ensured by the Fermi liquid of collective electrons, whose concentration is determined by the valence of atoms and the density of ions.

The polarization of the lattice is only performed by the Fermi electrons whose effective weight and speed are determined by their dispersion law rather than all collective electrons [24].

The minimum possible lattice polarization time is determined by the highest (Debye) frequency ω_*D*_ of atom oscillations and equals half of the ion oscillation period.

(13) $$T/2=\pi /\omega_{D}.$$

The lattice polarization area with a length of 2ξ = 2π × *v_F_*/ω_*D*_, featuring an excessive positive charge, moves behind it at a distance that exceeds atom spacing (which equals fractions of a nanometer) by many times.

Note that this polarization cannot be seen as the electron generation of acoustic or optical phonons propagating in the lattice as elastic waves. Thus, we can claim that the polarization of the lattice is associated with the appearance and disappearance of virtual (active for a very short time) phonons with a frequency close to the Debye frequency, i.e., in this case, the electron in the crystalline lattice is surrounded by a cloud of virtual phonons with the Debye frequency.

The greater the polarization, the more virtual phonons that are generated and the stronger the link between the electron and the lattice. The energy of the interaction between the electron and the lattice is determined by the constant λ of electron–phonon interaction. The value of λ can be expressed with the number of virtual phonons *ћ* × ω_*D*_ excited by the electron. This number represents the proportion of the lattice polarization (elastic deformation) energy *W_p_* in the polarization area and the phonon energy. The constant λ is defined as follows: (1/3 accounts for the existence of three phonon range branches)

(14) $$\lambda =\frac{1}{3}\times \frac{W_{p}}{\hbar \times \omega_{D}}.$$

Over time, τ = *a*/*v_F_* (*a* is the lattice constant), reflecting the interaction of the ion with mass *Μ* and a Fermi electron, and the ion impulse changes by the following value:

(15) $$\Delta p=F\times dt\approx \frac{e^{2}}{a^{2}}\times \frac{a}{v_{F}}=\frac{e^{2}}{a^{2}\times v_{F}}.$$

The polarization energy of one ion is as follows:

(16) $$W^{*}\approx \frac{\Delta p^{2}}{2\times M}=\frac{e^{4}}{a^{2}\times v_{F}^{2}\times 2\times M}.$$

The number of ions in the polarization area is as follows:

(17) $$N\approx 2\xi /a=2\pi \times v_{F}/(a\times \omega_{D}).$$

The energy associated with the elastic deformation of the lattice in the polarization area is as follows:

(18) $$W_{R}=\left|W^{*}\right|\times N=\left|W^{*}\right|\times \frac{2\pi \times v_{F}}{a\times \omega_{D}}\approx \frac{\pi \times e^{4}}{a^{3}\times v_{F}\times \omega_{D}\times M}.$$

To sum up, alternating electric potential excites oscillatory polaron generation in the conducting PM structure. These polarons are a combination of an electron and the elastic lattice deformation (polarization) field generated by it. As this electron moves, it always polarizes the lattice, and when it transits to the next elementary cell, polarization moves with it. Since polarization energy is preserved in this case, the associated electron + lattice polarization area (i.e., polaron) moves along the interaction of electrons and the lattice. The stronger the electron’s polarization of the latter, the greater the polarization area that moves along with the electron, and the greater the effective polaron mass. When this polaron moves, a magnetically ordered structure obtains the respective local magnetizing heterogeneity.

Thus, these quasi-particles in metal objects are essentially negatively charged polarons with a charge of −*e* and an effective mass of *m_P_*.

In the general case, the motion equation for the local magnetizing heterogeneity can be written down as follows [25]:

(19) $$\frac{dM}{dt}=-\gamma \times M\times H_{eff}+R,$$

where **R** is the dissipative term accounting for energy losses; **H***_eff_* is the effective magnetic field that is a functional derivative of the ferromagnetic energy *W* based on magnetic moment **M**(*x*, *t*).

(20) $$H_{eff}(r,t)=\frac{\delta W}{\delta M(x,\;t)}.$$

The conducted analysis of physical processes allowed for us to conclude that alternating electric potential forms an area of elementary polaron harmonic oscillators in the conducting PM structure whose radial oscillations can be described with the following expression:

(21) $$m_{Pi}\times \frac{d^{2}r_{i}}{dt^{2}}+m_{Pi}\times \beta \times \frac{dr_{i}}{dt}=-e\times E_{ri}\times \exp(j\omega_{0}t),$$

where β is the electron friction factor.

Considering the above, virtually all of the conducting PM structure enters the harmonic oscillator mode for PM magnetism.

**M***_i_*(**r***_i_*, *t*) = **M***_i_*(**r***_i_*, *t* + *nT*), (22)

where *T* is the regularity increment; *n* is an integer.

According to (22), this simple harmonic oscillator can be written down as follows:

(23) $$\frac{d^{2}M_{i}}{dt^{2}}-{\omega^{2}}_{s}\times M_{i}=Q(r_{i},t),$$

where $Q(r_{i},t)=-\frac{e\times E_{mi}}{m_{pi}}\times \sin(\omega_{0}t+\varphi_{E})$ is a function that characterizes distributed external impacts.

By solving this inhomogeneous second-order differential Equation (23) with due account of the resonant harmonic oscillator operation, the following expression is obtained:

(24) $$M_{i}=M_{mi}\times \cos(\omega_{0}t+\varphi_{0})=-\frac{e}{m_{e}\times V}\times s\times \cos(\omega_{0}t+\varphi_{0}).$$

Thus, the radial oscillatory movement of polarons perpendicular to the PM main field *H*_0_ initiates the link between various types of magnetism oscillations and spin waves that can lead to the so-called instability of specific spin wave types, i.e., their excitation. This results in the parametric excitation of spin waves or their parallel (longitudinal) pumping.

**Figure 3:**
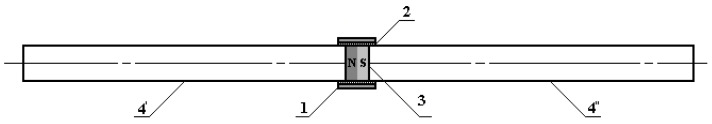
FRS Module.

Place a similar polaron harmonic magnetizing oscillator in the center of a simulated waveguide formed by two identical coaxial round dielectric ferrite rods (FR) 4′ and 4″ with a finite length (Figure 3). This set of elements is referred to as the ferromagnetic rod system (FRS).

Consider the specifics of physical processes that are possible in this FRS when a similar polaron harmonic oscillator is activated.

The theory of magnetically ordered objects considers not only the mutual positioning of atomic magnetic moments **P***_J_* in the material, i.e., their magnetic structure, but also the fluctuations of these moments due to various external factors (heat excitation, electromagnetic radiation, etc.). The simplest way of controlling the domain structure is by applying a magnetic field, as the magnetic domain structure induced by it is partially preserved after the field is down, leading to the emergence of a respective metastable structure.

Although this is prevented by exchange interactions, the selection of a specific factor that disrupts the magnetic order in the system of magnetic moments **P***_J_* can excite dynamic heterogeneities in magnetically ordered materials formed by magnons, quasi-articles representing a quantum of spin wave oscillations.

Following the particle-wave duality principle, the energy *W_M_* and moment momentum **s** of one magnon are as follows:

*W_M_* = *ћ* × ω, **s** = *ћ* × **k**, (25)

where *ћ* = h/2π, and h is the Planck constant.

Each magnon is a single inverted electronic spin (distributed across the entire FRS). Therefore, it reduces the overall magnetism of the FRS by the value of the magnetic moment directed opposite to its magnetism, as follows:

γ × *ћ* ≅ 2µ*_B_*, (26)

where $\gamma =\left|e\right|\times g_{s}/(2m_{e}\times c)$ is the magneto–mechanical ratio, *e* is the electron charge, *g_s_* is the spectroscopic splitting factor, *c* is the speed of light in vacuum, and µ*_B_* is the Bohr magneton.

Thus, the complete magnon-based measurement of the permanent FRS magnetism is as follows:

(27) $$M_{0}-M_{0}(t)=\sum_{k}\gamma \times \hbar \times n=M,$$

where *n* is the number of magnons in the state with wave vector **k**. The summing is conducted with all permitted values of *k* in the first Brillouin zone.

In other words, this impact results in magnetic (spin) waves and respective quasi-particles—magnons occurring in the FRS structure (similar to the lattice phonons), whose numbers characterize the intensity of sin waves and are proportional to the squares of their amplitudes.

In linear approximation, the evolution of this wave magnetism oscillation can be written down as follows:

(28) $$\frac{\partial^{2}M}{\partial x^{2}}-k^{2}\times \frac{\partial^{2}M}{\partial t^{2}}=0,$$

where *k* is the wave propagation factor.

**Figure 4:**
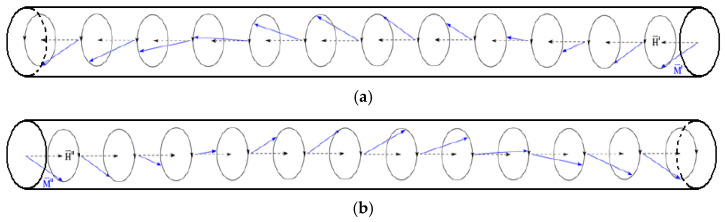
The distribution of spin waves in the elements of FRS: (**a**)—in dielectric FR 4′; (**b**)—in dielectric FR 4″.

Note that the activation mode of the polaron harmonic oscillator is selected so that coherent spin oscillations with defined ω and **k** and ordered phases are excited in the FRS on the spin wave resonance frequency. They provide the generation (emergence) of multi-directional circular-polarized spin waves running from the polaron harmonic oscillator as cone-type helices (Figure 4).

(29) $$\mathrm{for}\;\mathrm{FR}\;4^{\prime }:\;M^{\prime }(x,t)=M_{m}^{\prime }\times \cos\left(\omega t-k\times x+\varphi \right);\;\mathrm{for}\;\mathrm{FR}\;4^{\prime \prime }:\;M^{\prime \prime }\left(x,t\right)=M_{m}^{\prime \prime }\times \cos\left(\omega t+k\times x+\varphi \right),$$

where **k** = *k* × **n** is the wave vector directed along normal n to the wave surface, whose length equals wave number *k* = 2π/λ = ω/*v*.

## 3. Development of a Fluxgate Magnetometer Based on a New Excitation Principle

**Figure 5:**
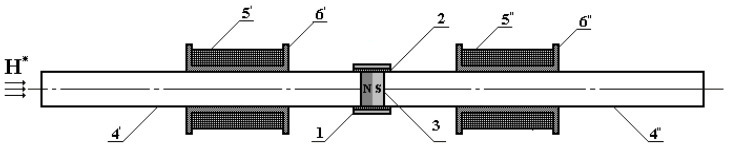
FM prototype.

Place a coaxial FRS inside two compensated cylindrical input measurement coils, *MC*_1_ and *MC*_2_, each of which is made of electrical winding 5′ and 5′′ located on respective dielectric frames 6′ and 6′′ (Figure 5). In this case, this set of elements can be seen as a prototype of a fluxgate magnetometer (FM) whose FRS structure is axially impacted by the measured permanent magnetic field H^*^.

In this case, EMF is excited in electric windings 5′ and 5″ of measurement coils 6′ and 6″, respectively, that feature aligned activation due to the periodic changing of magnetism **M** at a certain frequency ω. This happens at the same frequency ω or the frequency of one of the higher harmonics *k* × ω, proportional to *dM*/*dt*, i.e.,

(30) $$e_{s}^{\prime }(t)=C\times \frac{dM^{\prime }}{dt}—\mathrm{for}\;\mathrm{FR}\;4^{\prime }\;e_{s}^{\prime \prime }(t)=C\times \frac{dM^{\prime \prime }}{dt}\;\mathrm{for}\;\mathrm{FR}\;4^{\prime \prime }$$

where *C* is the respective link constant.

Note that the initiation of an indirect oscillating electron exchange between ferromagnetic areas of FRS may result in co-linear ferromagnetic magnetizing ordering or co-linear anti-ferromagnetic ordering in adjacent magnetic areas. In this case, the structure with magnetic ordering changing from area to area can be seen as a system of magnetic domains. Inside each domain magnetism is parallel or anti-parallel, depending on the exchange type. The emergence of these domain borders results in the mixing of spin states of electrons and has a large impact on the magnetic resistance of magnetically ordered structures, which ultimately allows for controlling the magnetic permeability of magnetically ordered structures.

In this case, the measured permanent field H^*^, which is axial to the FRS, is modulated due to the periodic changing of magnetic permeability μ of the FRS material, i.e., a μ transformation is performed due to the magnetic modulation effect (MME).

This μ transformation mode is essential for an FM operating mode with longitudinal excitation. Thus, according to the existing parametric theory of flux gates with longitudinal excitation that uses the Taylor series’ expansion of the function *B*(*H*_Σ_) with *H*_Σ_ = *H*_1_ + *H*_0_, we can write the following for minor impacts:

(31) $$B_{0}(t)=2\times \mu_{0}\times \mu^{*}(H_{1})\times H_{0}(t).$$

Note that with the given excitation field intensity amplitude, the function $\mu^{*}(H_{1})$ can be viewed as a time function $\mu^{*}(t)$.

For the physical processes studied, modulation is understood as the changing of the magnetic permeability of the FR 4′ and FR 4′′ material when it is affected by physical fields.

Considering the above, we can write the following:

(32) $$\mu^{*}(t)=\mu_{p}\times [1+m_{MM}\times \cos\omega_{p}t],$$

where $m_{MM}=\eta_{MM}\times H_{1m}$ is the magnetic modulation depth factor; η*_MM_* is the magnetic modulation transformation factor; *H*_1*m*_ is the intensity amplitude of the magnetic modulation field; and ω_*P*_ is the cyclic resonant excitation frequency of transformation.

Note that the cyclic resonant transformation frequency can only be determined in experiments during the registration of the maximum signal value at the FD output. Thus, when a specific FD unit of the type in question is used in practice, the transformation process excitation frequency is determined individually in experiments.

Expressions (31) and (32) show that the measured permanent magnetic field H^*^, directed axially to FR 4′ and FR 4″, uses the parametric modulation of oscillating magnetic permeability of the ferromagnetic material of FR 4′ and FR 4″ to transform into an alternating magnetic field with respective induction.

(33) $$B_{0}(t)=\mu_{0}\times \mu^{*}(t)\times H_{0}.$$

The variations of the measured magnetic field caused by magnetic permeability modulation processes affect windings 5′ *MC*_1_ and 5″ *MC*_2_ and induce the respective EMF in them, as follows:

(34) $$e_{M}^{\prime }(t)=-S_{1}\times w_{1}\times \frac{dB_{0}(t)}{dt}\mathrm{and}\;e_{M}^{\prime \prime }(t)=-S_{2}\times w_{2}\times \frac{dB_{0}(t)}{dt}.$$

By inserting (33) into (34), we obtain the following data for each measurement coil:

for *MC*_1_,

(35) $$\begin{array}{l} e_{M}^{\prime }(t)=2\times S_{1}\times w_{1}\times \mu_{0}\times \left[\mu^{*}(t)\times \frac{dH_{0}}{dt}+H_{0}\times \frac{d\mu^{*}}{dt}\right]=-2\times S_{1}\times w_{1}\times \mu_{0}\times H_{0}\times \frac{d\mu^{*}}{dt}=\\ =2\times S_{1}\times w_{1}\times \mu_{0}\times \mu_{p}\times \omega_{p}\times H_{0}\times m_{MM}\times \sin\omega_{p}t=\\ =2\times S_{1}\times w_{1}\times \mu_{0}\times \omega_{p}\times H_{0}\times \eta_{MM}\times H_{1m}\times \sin\omega_{p}t; \end{array}$$

for *MC*_2_,

(36) $$\begin{array}{l} -e_{M}^{\prime \prime }(t)=2\times S_{2}\times w_{2}\times \mu_{0}\times \left[\mu^{*}(t)\times \frac{dH_{0}}{dt}+H_{0}\times \frac{d\mu^{*}}{dt}\right]=-2\times S_{2}\times w_{2}\times \mu_{0}\times H_{0}\times \frac{d\mu^{*}}{dt}=\\ \;\;\;\;\;\;=2\times S_{2}\times w_{2}\times \mu_{0}\times \omega_{p}\times H_{0}\times (m_{MM}+m_{AM})\times \sin\omega_{p}t=\\ \;\;\;\;\;=2\times S_{2}\times w_{2}\times \mu_{0}\times \omega_{p}\times H_{0}\times \eta_{MM}\times H_{1m}\times \sin\omega_{p}t. \end{array}$$

where *S* = *S*_1_ = *S*_2_ are the section areas of FR 4′ and FR 4″; *w* = *w*_1_ = *w*_2_ is the number of turns in windings *MC*_1_ and *MC*_2_.

To sum up, using expressions (30) and (35), as well as (36) for a differential signal from the FD output in the field meter mode, we can write down the following:

(37) $$e_{\Sigma }(t)=[e_{M}^{\prime }(t)+e_{S}^{\prime }(t)]-[-e_{M}^{\prime \prime }(t)+e_{S}^{\prime \prime }(t)]=2e_{M}(t).$$

**Figure 6:**
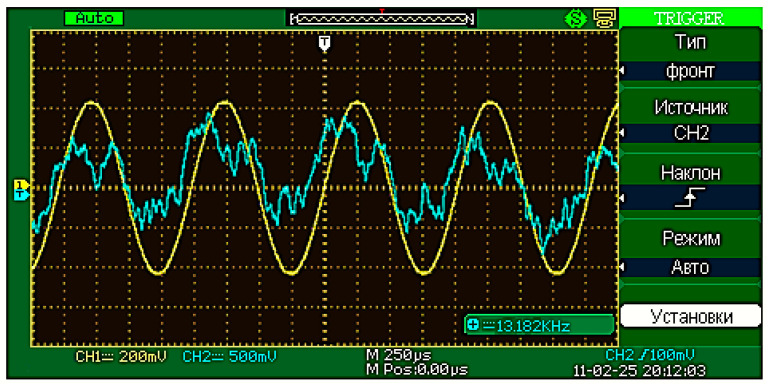
The oscillograms of the differential FM output signal: blue—output data signal following bandpass filtering and amplification (if measured magnetic field is present); yellow—output data signal following selective filtering (if measured magnetic field is present).

During experimental research with a measured magnetic field, we obtained the oscillograms of the differential FM output signal after its primary processing (bandpass filtering, amplification, and subsequent selective filtering) that reflected its waveshape and frequency. These oscillograms are shown in the Figure 6.

**Figure 7:**
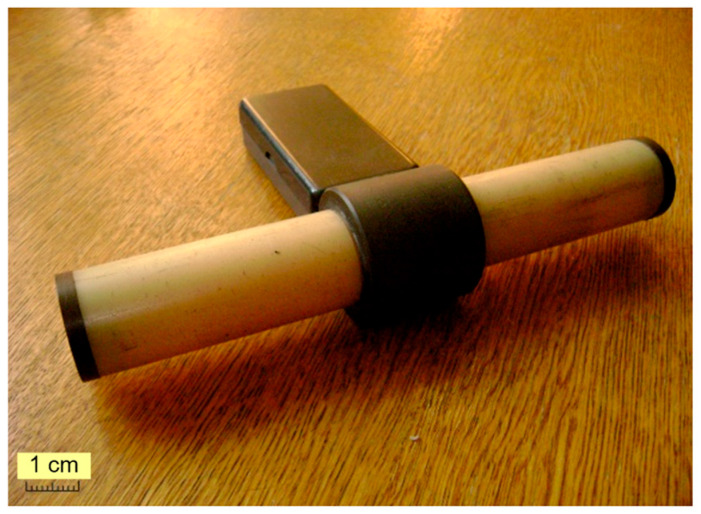
Overview of the new FM option.

The obtained analytical expression (37) shows that the usage of the exchange interaction effect creates the possibility of registering the measured permanent magnetic field with an FM that operates based on the new physical principle. The advantages of the suggested FM option shown in Figure 7 include its design simplicity, performance, interference immunity, transformation precision, and very low power consumption.

For a more convincing interpretation of the suggested solution, we used the physical effects (PE) theory to develop a flow chart for the PE in question that is normally manifested in several main action results that, in turn, depend on several additional impact parameters. Thus, it became possible to synthesize complex structures of interrelated PE facilitating the implementation of the research object (FM in this case) with several inputs and outputs, feedback, and the usage of PE complexes for the implementation of frequent functions. In this case, the combination of PE represents the physical structure of the object (PSO) that provides an idea on which set input parameters of the considered object are transformed into the set output parameters. The PSO elements include physical objects that experience PE and links between PSO elements that characterize the types of physical value transformations.

The studied FD option is also a complex hierarchic system characterized by a multitude of structural elements and links between them. The operation of this system is based on several key interlinked physical effects that are represented in a generalized way in Figure 7, where MC_1_ and MC2 are measurement coils of the first and second FD semi-elements, respectively; ECon are conductivity electrons of the PM material; CLN are crystalline lattice nodes of the PM material; DS_1_ and DS_2_ are domain systems of the FR 4′ and FR 4″ material, respectively; DS_0_ is the domain system of the PM material; EMF_1_ and EMF_2_ are the induction electromotive forces of MC_1_ and MC_2_, respectively; PMMF is the magnetic field of the permanent magnet; MMF is the measured permanent magnetic field; EFP is the alternating electric field potential; AMF_1_ and AMF_2_ are alternating magnetic fields in FR 4′ and FR 4″, respectively; FF′ and FF″ are force fields of different levels that occur as physical effects in crystalline structures of PM; SW_1_ and SW_2_ are spin waves initiated in FR 4′ and FR 4″, respectively; RSW_1_ and RSW_2_ are the running spin waves in FR 4′ and FR 4′; and PF is the polaron field.

**Figure 8:**
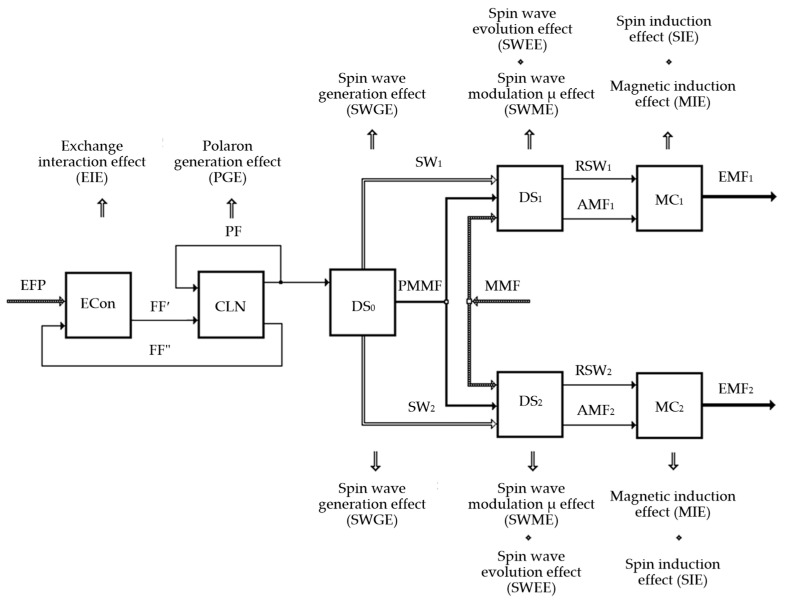
Physical effects flowchart.

Note that the ECon, CLN, and DS_0_ shown in Figure 8 are the structural elements made of the PM material, while DS_1_ and DS_2_ are the structural elements made of the FR 4′ and FR 4″ materials that form FRS.

When the ECon made of PM is affected by the EFP, the presence of EIE helps initiate the respective physical force field FF′ interacting with the CLN made of PM. This interaction results in the spatial displacement of the CLN that, in turn, generates a physical force field FF″ that also affects the ECon made of PM. The organization of similar interrelated processes results in the formation of polarons whose fields (PF) affect the CLN made of PM, respectively. This physical process with a complex hierarchy results in a PGE that can be observed as stable elementary polaron harmonic oscillators within the structure of the PM material. The PF generated in this case affects the DS_0_ made of PM, resulting in the initiation of the SWGE in it. Spin waves SW_1_ and SW_2_ that occur in this case evolve (SWEE) to become DS_1_ and DS_2_ that are magnetically ordered by the PMMF, and are additionally magnetized by the measured permanent magnetic field MMF, causing the SWME to occur in them and resulting in the formation of alternating magnetic fields AMF_1_ and AMF_1_ in DS_1_ and DS_2_. Subsequently, the running spin waves AMF_1_ and AMF_1_ that occur in DS_1_ and DS_2_ as a result of the evolution of spin waves SW_1_ and SW_2_, as well as alternating magnetic fields AMF_1_ and AMF_1_, induce the respective total EMF_1_ and EMF_2_ in MC_1_ and MC_2_ turns (SIE and MIE) that become the output signal of the FD.

The flowchart of physical effects shown in Figure 8 provides a complete representation of the set input parameters that are transformed into the set output parameters, i.e., present the operating function of the FM, while the charts of specific physical effects reflect the physical bases of the FM and demonstrate the functional links between its structural elements.

The operation of the considered FM is based on the developed PE flowchart that confirms the possibility of a physical implementation of a new FM type with the exchange interaction effect.

## 4. Experimental Research of FM Based on a New Principle

During the development of a measurement device (MD), it is crucial to assess its efficiency. This assessment can be performed if impact factor parameters in the operating area of the MD and its output signal parameters are known. Knowing these parameter values helps to relatively accurately determine the required dimensions of the developed device.

The organization of research and the determination of various properties of the developed MDs based on new operating principles, including the considered FM that features specific properties that make it versatile and highly applicable, are associated with certain problems.

Measuring physical parameters like magnetic field intensity requires complex equipment and is not always possible due to background interference. However, there are certain methods that can ensure artificial conditions for quick and direct measurement of FM parameters, e.g., through the standard field layout (SFL) (Helmholtz field). The obtained magnetic field is heterogeneous, and its intensity may be measured by simple tools. In this case, SFL is used as a reference for the reproduction of the magnetic induction value with the set error rating.

In some cases, it is necessary to generate a magnetic field with a specific intensity inside the magnetosensitive element of the FM. The SFL used in this situation is generally based on coils, inducing a magnetic field. To generate a homogeneous field inside the magnetosensitive element of the FM, these coils are placed at a large distance from it. The drawbacks of this induction measure generation method include a large coil weight and power consumption, the need for coil circuit thermocompensation, etc.

**Figure 9:**
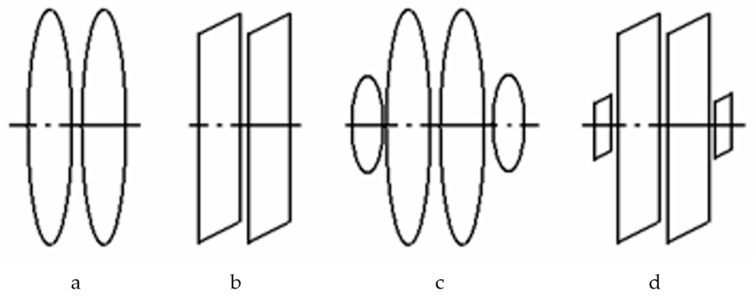
Various geometric configurations of rings.

A more rational SFL generation method is based on the usage of Helmholtz rings (HR) located next to the magnetosensitive element [26]. Conventionally, these problems are solved using the following HR geometry options: a pair of identical round rings (Figure 9a), a pair of identical square rings (Figure 9b), and two ring couples (Figure 9c,d).

The conducted analysis showed that, in terms of power consumption and generated field homogeneity, the best option is the one where all rings have an identical size and geometry, making for the first or second configuration.

**Figure 10:**
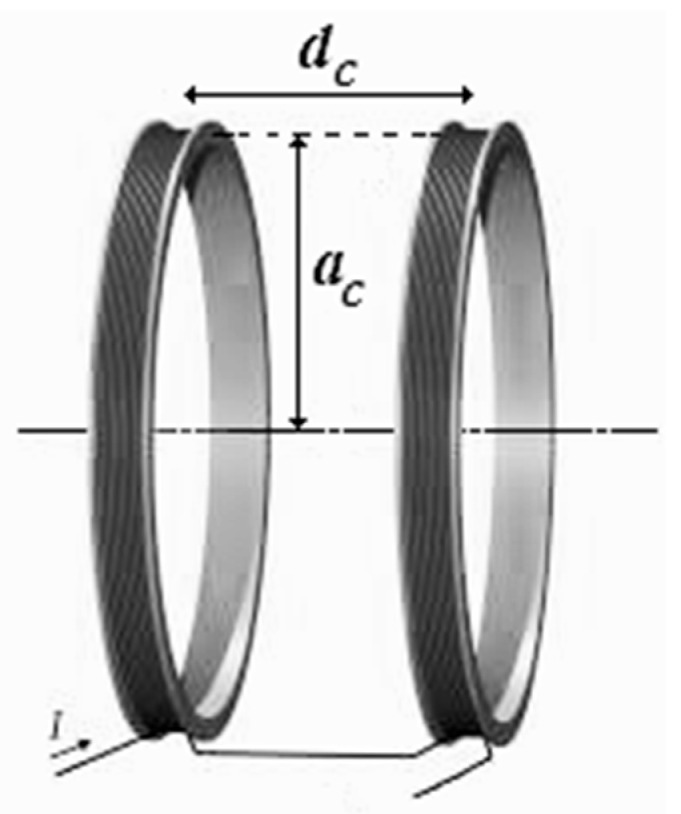
Round rings.

Note that optimal geometry can be achieved with round rings. With this configuration, round coils have smaller sizes compared to square ones when the extents of homogeneous fields generated by them are equal. Round rings are also optimal in terms of energy. Thus, the best geometry configuration of all of the possible ones is the one with non-repeated round rings (Figure 10).

Note the HR radius as *a_c_* and the distance between HR centers as *d_c_*, and assume *d_c_*/*a_c_* = 1.116, which effectively corresponds to the optimal geometry configuration. The rated field for an HR pair is the field generated by them on their axis halfway between their centers. Its intensity can be calculated using the following formula:

(38) $$H=\frac{N\times I}{a_{c}\times {\left({\left(\frac{d_{c}}{2a_{c}}\right)}^{2}+1\right)}^{\frac{3}{2}}},$$

where *N* × *I* is the number of ampere-turns in each HR.

**Figure 11:**
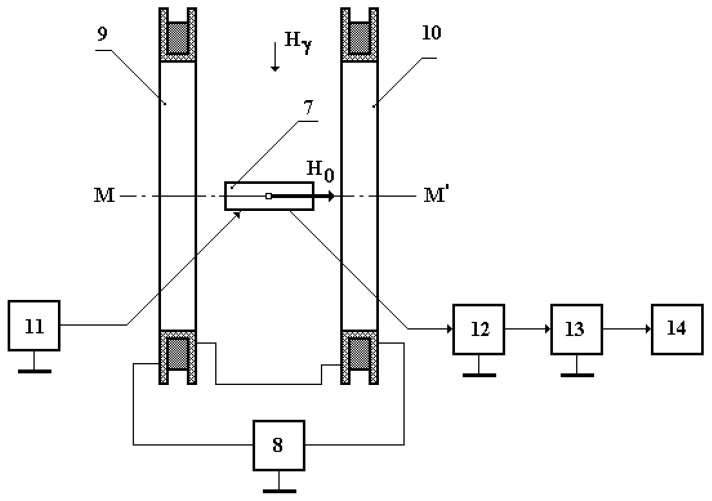
The structure flowchart of the TB.

Based on the selected SFL, we developed a test bench (TB) (Figure 11) for the laboratory testing of the new FM option, which includes the following functional components: 7—FM layout option; 8—controlled direct current source with galvanic decoupling from the ground; 9 and 10—Helmholtz rings (HR) (controlled source of heterogeneous permanent magnetic field); 11—electric charge generator with alternating electric field potential; 12—measuring module; 13—software–hardware interface; 14—calculation module (PC); *H*_γ_—background geomagnetic field; M-M′—HR symmetry axis perpendicular to the direction of the vector of harmonic component *H*_γ_ of the background geomagnetic field at the TB location; and *H*_0_—magnetic field generated by the SFL along the spatial axis M-M′.

With the said mutual spatial positioning of HR and the vector of horizontal component *H*_γ_ of the background geomagnetic field, we can consider FM 7 sufficiently immune to the background geomagnetic field *H*_γ_.

Review the operating principle of the TB in the general case.

Helmholtz rings 9 and 10 and the controlled DC source 8 generate a local uniform field SFL *H*_0_, imitating the measured magnetic field at the spatial location of FM 7 along its spatial axis M-M′.

The alternating potential of the electric charge field of generator 11 initiates the excitation of the FM, which results in the FM switching to the operating mode.

When the local SFL *H*_0_ is applied to FM 7, its output induces an electrical data signal registered by the metering module 12. Following the respective transformation (filtering and amplification) in the metering module 12, the electrical data signal is sent through the software–hardware interface 13 to the calculation module 14 that performs the required algorithmic treatment and the subsequent definition of the parameters of the SFL *H*_0_, imitating the measured magnetic field.

Adjusting the DC source 8 helps adjust the value of SFL *H*_0_ to simultaneously register the respective FM response to these changes, which ultimately helps to research the main characteristics of the suggested FM option.

To facilitate the changing of the magnetic field intensity vector module and direction on the TB, we determined the geometric and electrical parameters of HR rings over a wide range based on the following requirements: 1—the field must be homogeneous in the sensor prototype area; 2—assume that a 10-cm spherical area is enough for experimentation; 3—the heterogeneity degree of the field intensity module is understood as the ratio of intensity module deviation from the rated value to the rated value of $\delta_{m}=(B-B_{0})/B_{0}$; and 4—the heterogeneity degree of the direction is understood as the angle between the intensity vector of the rated field and that of the actual field at point $\delta_{a}=\arccos(B_{0}/B)$.

These requirements can be met if the working area satisfies conditions δ*_m_* < 1% and δ*_a_*< 0.5°. There is no point in setting a lower field heterogeneity, as the stated values can be compared to the errors of standard magnetometers.

The TB uses the SFL to generate a magnetic field following a preset rule for its parameter changes (intensity module and direction), and the FM is placed into it. Based on the FM response to the active SFL magnetic field, we can make conclusions about the FM metrological parameters.

**Figure 12:**
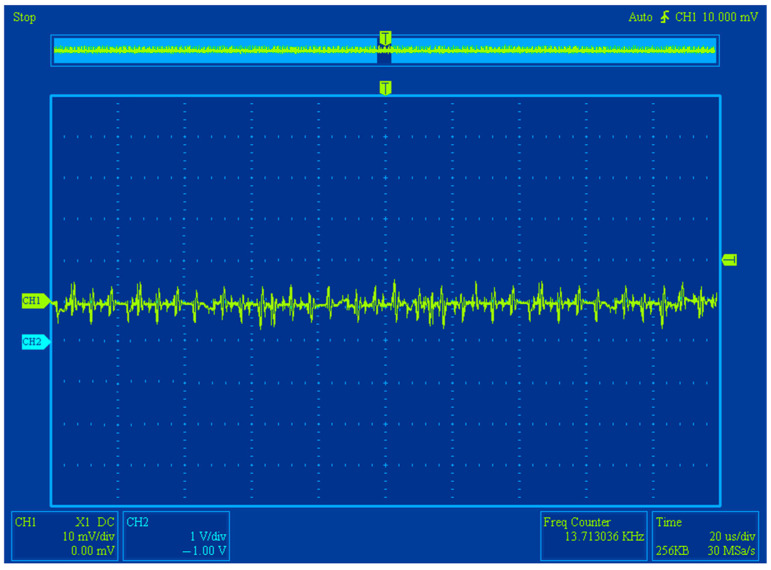
FM output signal oscillograms when there is no *H*_0_.

**Figure 13:**
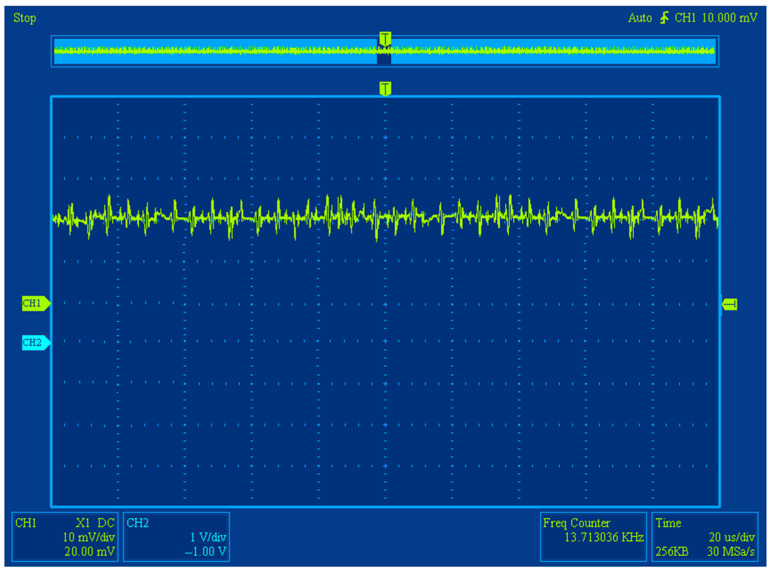
FM output signal oscillograms when under *H*_0_.

The examples of the obtained FM output data signal oscillograms with and without an external field are shown in Figure 12 and Figure 13, respectively. The oscillogram shows the output data signals of the FM after secondary processing (amplitude detection).

**Figure 14:**
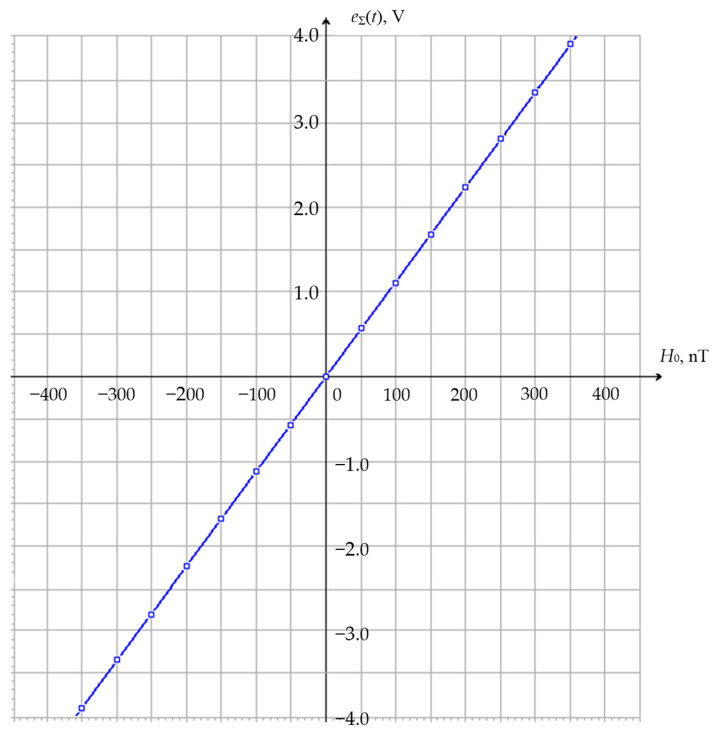
Static FM characteristic.

The experiments conducted on the TB resulted in a static FM characteristic representing a dependency between the electrical parameter of the FM (output emf) and the measured non-electric value (Helmholtz magnetic field intensity) (Figure 14).

**Figure 15:**
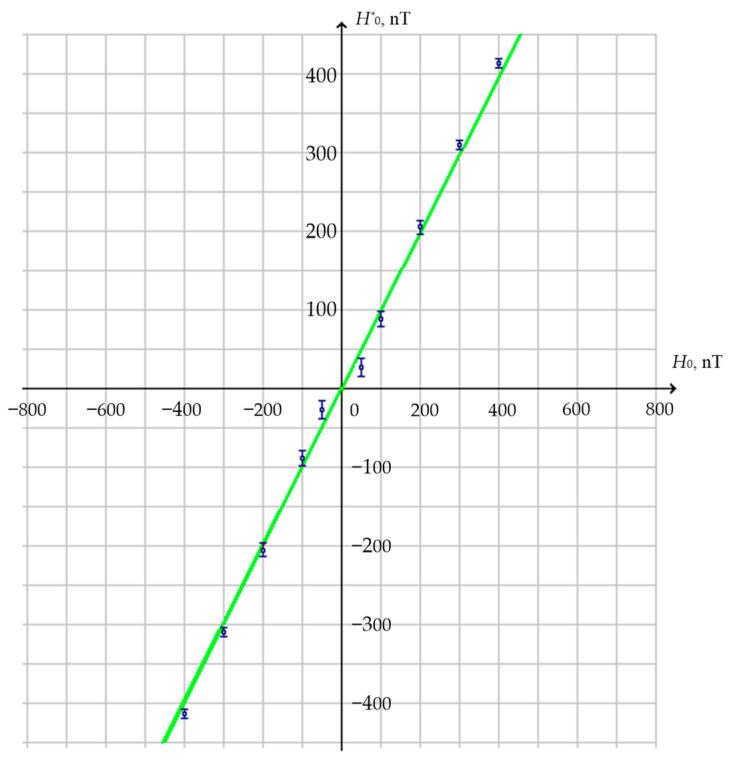
FM characteristic reflecting the accuracy of FM transformation of the measured non-electric input value.

The FM characteristic reflecting the accuracy of FM transformation of the measured non-electric input value is shown in Figure 15, where the green straight line reflects the rated value trend for the impacting (measured) field *H*_0_, while the points with measurement errors marked reflect the measurement results for H^*^_0_ of the impacting field *H*_0_.

Experimenting on the developed TB, we determined the following key FM parameters in the field meter mode: measurement range ± 40 μT; detection threshold 0.5 nT; sensitivity 0.05 V/nT; key permitted measurement error ± 10 nT; power voltage 5 V; and consumed power 50 mW. Low power consumption can be explained by the fact that all of the main physical effects that determine the operating principle of the FD are initiated by the variations in the electric charge field potential rather than the electric current. Thus, the FD consumes a few milliamperes of power in the resonant mode. In addition, the metering windings of the FD loaded with high-resistance input circuits of the subsequent transformation elements generate electric voltage and not current. The low overall electric current consumption with a relatively low FD excitation voltage (around 5 volts) is the reason why the consumed power is around 50 mW.

## 5. Conclusions

To rationalize the suggested new construction principle of the fluxgate magnetometer (FM), we used the following phenomenological (descriptive) approach, which primarily solved the problem of ordering and the scientific generalization of experimental data: it exposed the connections between phenomena and their properties, physical mechanisms behind the phenomena and processes in question, and significant links between them using the basic provisions of well-known laws of physics [27].

This paper demonstrated that the usage of a PM made of a composite-conducting material with a complex crystalline lattice structure and featuring an indirect exchange interaction initiates the formation of polarons with oscillating magnetism when this structure is subjected to an electric charge with alternating potential. This effect causes significant changes in the entropy of the indirect exchange interaction and the associated sublattice magnetism oscillations, which ultimately leads to the generation of spin waves. The use of ferrite rods made of a dielectric material as waveguides is a channeling factor in the evolving propagation of running spin waves that have a macro-level electrodynamic impact on the measurement coils of the FM and perform the parametric modulation of magnetic permeability of waveguide material, which results in the formation of the respective measured magnetic field variations.

In terms of design and technology, the FM with a new excitation method has advantages over conventional flux gates that require a multi-turn electrical winding of the excitation coil. The alternating current passing through the multi-turn coil winding causes it to heat, which is a significant destabilizing factor for flux gate operation. In addition, the requirement to use a multi-turn coil brings up certain technological problems in FM production, restrictions to exciting magnetic field parameters (intensity amplitude and frequency), and causes a significant increase in size, weight, and costs. The use of a cylindrical metal electrode as a source of the exciting electrical charge with variable electrical potential mitigates the mentioned drawbacks of conventional flux gates, thus giving the new type of flux gate a competitive edge.

Thus, the described FM excitation occurs if the following three obligatory conditions are fulfilled: 1—it is magnetically ordered, conducting permanent magnet material; 2—it has a composite, non-conducting, and ferromagnetic FM working rod material; and 3—the excitation source (e.g., a cylindrical metal electrode) has an alternating potential. The absence of at least one of these becomes a factor that prevents the initiation of working processes in the FM as a whole.

Based on the analysis of the described physical processes that were not previously used in the FM operation, we obtained analytical expression (37), which demonstrates that the usage of exchange interaction allows for the registration of the measured permanent magnetic field through the registration of the EMF generated in the metering coils. The validity of this expression has been fully confirmed in experiments. Based on the above, we can claim that the operation of the suggested FM is based on a new physical principle that was not previously used in FMs. Thus, the developed phenomenological theory of the proposed FM can be deemed valid because it helped order and generalize the physical phenomena that occur in the FM (see Figure 7 for the logical sequence) and allowed for the numerical assessment of the FM response to a measured external magnetic field.

In this case, the usage of the phenomenological approach can be justified by the fact that there is currently no established theory or comprehensive mathematical description of the physical effects of non-uniform alternating electric field potential applied to the oscillatory nature of conduction electron spin polarization in metal and magnetically ordered structures. This paper constitutes the first step in the study and theoretical explanation of this effect that was discovered in experiments and used to develop a brand-new FM type.

Note that this paper presents the first stage of the research on the development of fluxgate magnetometers using a new excitation principle. The goal of this stage was to develop and theoretically justify the physical effects of the alternating potential of a heterogeneous electric field on the oscillatory nature of conduction electron spin polarization in metal and magnetically ordered structures, as well as the possibility of using these effects in FM. We established that the initiation of the oscillating indirect exchange of electrons between the ferromagnetic areas of the core ferrimagnetic system of the FM provides an opportunity to control their magnetic permeability. This μ-transformation mode is essentially an operating mode of the FM with longitudinal excitation. The proposed theoretical explanation for the processes occurring in the suggested FM was confirmed in experiments.

The goal of the second stage of the research shall include the development of the optimal hardware implementation of fluxgate magnetometers with the proposed excitation principle, its extended experimental research, including the detailed study of resonant operating modes, and the comparison of operating properties and capabilities of the proposed FM and similar existing equipment. This second stage is a standalone piece of research that requires significant efforts, and combining it with the first stage is deemed unfeasible.

Based on the suggested FM excitation method and the basic design of its implementation device, a wide array of opportunities arise for the research and development of various-purpose magnetometric equipment operating based on new physical principles.

## Data Availability

The original contributions presented in this study are included in the article. Further inquiries can be directed to the corresponding author(s).
